# *CrWSKP1*, an *SKP1-like* Gene, Is Involved in the Self-Incompatibility Reaction of “Wuzishatangju” (*Citrus reticulata* Blanco)

**DOI:** 10.3390/ijms160921695

**Published:** 2015-09-09

**Authors:** Peng Li, Hongxia Miao, Yuewen Ma, Lu Wang, Guibing Hu, Zixing Ye, Jietang Zhao, Yonghua Qin

**Affiliations:** 1 State Key Laboratory for Conservation and Utilization of Subtropical Agro-Bioresources/Key Laboratory of Biology and Genetic Improvement of Horticultural Crops-South China, Ministry of Agriculture, College of Horticulture, South China Agricultural University, Guangzhou 510642, China; E-Mails: ziyue7766@163.com (P.L.); hxmrain@163.com (H.M.); pinganma@126.com (Y.M.); wxiaolu0217@163.com (L.W.); guibing@scau.edu.cn (G.H.); qyh6k@163.com (Z.Y.); zhaojietang@gmail.com (J.Z.); 2 Institute of Tropical Bioscience and Biotechnology, Chinese Academy of Tropical Agricultural Sciences/Key Laboratory of Tropical Crop Bioscience and Biotechnology, Ministry of Agriculture, Haikou 571101, China

**Keywords:** *Citrus reticulata* Blanco, self-incompatibility, *SKP1*, genetic transformation

## Abstract

Plant S-phase kinase-associated protein 1 (*SKP1*) genes play crucial roles in plant development and differentiation. However, the role of *SKP1* in citrus is unclear. Herein, we described a novel *SKP1-like* gene, designated as *CrWSKP1*, from “Wuzishatangju” (*Citrus reticulata* Blanco). The cDNA sequence of *CrWSKP1* is 779 base pairs (bp) and contains an open reading frame (ORF) of 477 bp. The genomic sequence of the *CrWSKP1* gene is 1296 bp with two exons and one intron. *CrWSKP1* has high identity with *SKP1-like* genes from other plant species within two conserved regions. Approximately 85% of pollen tubes of self-pollinated *CrWSKP1* transgenic tobaccos became twisted at four days after self-pollination. Pollen tube numbers of self-pollinated *CrWSKP1* transformants entering into ovules were significantly fewer than that of the control. Seed number of self-pollinated *CrWSKP1* transformants was significantly reduced. These results suggested that the *CrWSKP1* is involved in the self-incompatibility (SI) reaction of “Wuzishatangju”.

## 1. Introduction

Seedless fruits can be of great value for consumers and the processing industry. Self-incompatibility (SI) is an important factor that can result in seedless fruits in citrus [1,2,3,4,5,6,7]. SI systems are divided into sporophytic SI (SSI) and gametophytic SI (GSI). In the SSI system of Brassicaceae, S-locus cysteine-rich protein (SCR)/S-locus protein-11 (SP11) and S-locus receptor kinase (SRK) were identified to regulate a signal transduction cascade in the stigmatic papillae [8]. In the GSI system of Rosaceae, Solanaceae, and Scrophulariaceae, SI operates as a complex process involving multiple factors. GSI system can be divided into S-RNase-based SI and *Papaver* SI. S-RNase-based SI is mainly controlled by the pistil *S* gene, encoding a ribonuclease (S-RNase), the pollen S-locus-encoded F-box protein (SLF/SFB) [9,10], and non-S-factors such as S-phase kinase-associated protein 1 (SKP1), *S1 self–incompatibility locus–linked pollen 3.15*, *ubiqutitn–activating enzyme E1*, and *Rho–like GTPase* genes [11,12,13,14,15,16,17,18]. *Papaver* SI is controlled by pistil *S*-determinant *PrsS* (*Papaver rhoeas* stigmas *S* determinant) interacted with a Ca^2+^-dependent signaling network resulting in programmed cell death (PCD) of “self” pollen [19,20].

SKP1 gene encodes a small protein of approximately 160 amino acids. Plants have a large number of *SKP1-like* genes and they play key roles in plant cell division, growth and flower development, and protein degradation. It has been shown that the pleiotropic functions of *SKP1* genes are consistent with their unique expression patterns for the respective genes in vegetative and reproductive tissues. Porat *et al.* [21] reported that the *ATskp1* gene is highly correlated with meristem activity and can be a marker for cells undergoing division in that its mRNA accumulated in all of *Arabidopsis* meristems and organ primordia. The *SKP1-like* family of *Arabidopsis* also exhibits a high degree of differential gene expression and gene product interaction during development [22,23,24]. *CgSKP1*, a novel *SKP1* gene from “Shatian” pummelo (*Citrus grandis* Osbeck), may play a vital role in flower development of “Shatian” pummelo since it was highly expressed in anthers, and ovaries, but lowly in styles, while it was gradually increased during flower development of “Shatian” pummelo [25]. Hong *et al.* [26] isolated six *SKP1-like* (*TaSKP*) genes from common wheat (*Triticum aestivum*) and suggested that they may be involved in various growth and flower developmental processes according to their expression patterns. Further, Yang *et al*. [27] suggested that the *ASK1* gene is essential for male meiosis and may control homologue separation. SKP1 and SKP1-related proteins can regulate the specific degradation of target proteins and affect the formation of other protein complexes in *Arabidopsis* [22].

The ubiquitination and 26S proteosome system (U/26S) is a major protein degradation pathway involving in plant pollen germination, pollen tube elongation and SI response. Skp1 is a core component of the SCF (Skp1, Cullin1 and F-box) complex mediating protein degradation by the U/26S. Sijacic *et al.* [28] demonstrated that *PhSSK1* as a component of the SCF complex encodes pollen factors in SI reaction. In lily (*Lilium longiflorum*), pollen-specific SKP1-like proteins are components of functional SCF complexes and essential for pollen tube elongation in GSI lily [29]. Pollen-expressed *SKP1-like* genes are probably involved in the SI reaction of plants. SLF-interacting *SKP1-like1* (*AhSSK1*) is expressed specifically in pollen of *Antirrhinum hispanicum* and acts as an indispensable player involved in SI response, for its adaptor role of associating AhSLF with CUL1 [11,12]. Matsumoto *et al.* [30] identified a Skp1-like protein interacting with SFB (PavSSK1) from SI sweet cherry (*Prunus avium*) using a yeast two-hybrid screening against the pollen cDNA library. *PavSSK1* was expressed strongly in anthers and pollen, weakly in styles, and not at all in other floral organs or leaves in sweet cherry, suggesting *PavSSK1* participated in the SI reaction of sweet cherry. These results suggest that *SKP1-like* genes operate as complex and multiple functions in pollen tube elongation and SI reaction. However, the role of *Skp1-like* gene in the SI reaction of citrus is unknown.

“Wuzishatangju” (*C*. *reticulata* Blanco) is a mutant originated from self-compatible (SC, seedy) cultivar “Shatangju” [31]. It is one of the most popular mandarin cultivars in China due to its seedless, very tasty and easy-to-peel characteristics. Cytological analyses showed that the seedlessness of “Wuzishatangju” results from GSI that blocks fertilization in the ovary [1]. Our previous studies showed that expression levels of *CrWSKP1* gene were significantly up-regulated in ovaries of “Wuzishatangju”, and approximately 20 times higher than that of “Shatangju”. The highest expression levels of *CrWSKP1* gene was detected in pistils at 4 days after self-pollination of “Wuzishatangju”, compared to the lowest expressions in pistils at 4 days after cross-pollination of “Wuzishatangju”×“Shatangju” [32] which is consistent with the cytological analyses [1]. We therefore hypothesized that the *CrWSKP1* gene is involved in the SI reaction of “Wuzishatangju” by U/26S pathway [33]. In this study, we isolated full-length cDNA and DNA sequences of *CrWSKP1* genes from SI “Wuzishatangju” mandarin based on its expressed sequence tags (ESTs) [32]. Functional characteristics of *CrWSKP1* were elucidated by transgenic experiments in tobacco. The aim of this study is to explore the role of *SKP1-like* gene in the SI response in citrus.

## 2. Results

### 2.1. Cloning and Sequence Analysis of SKP1-like Gene

**Figure 1:**
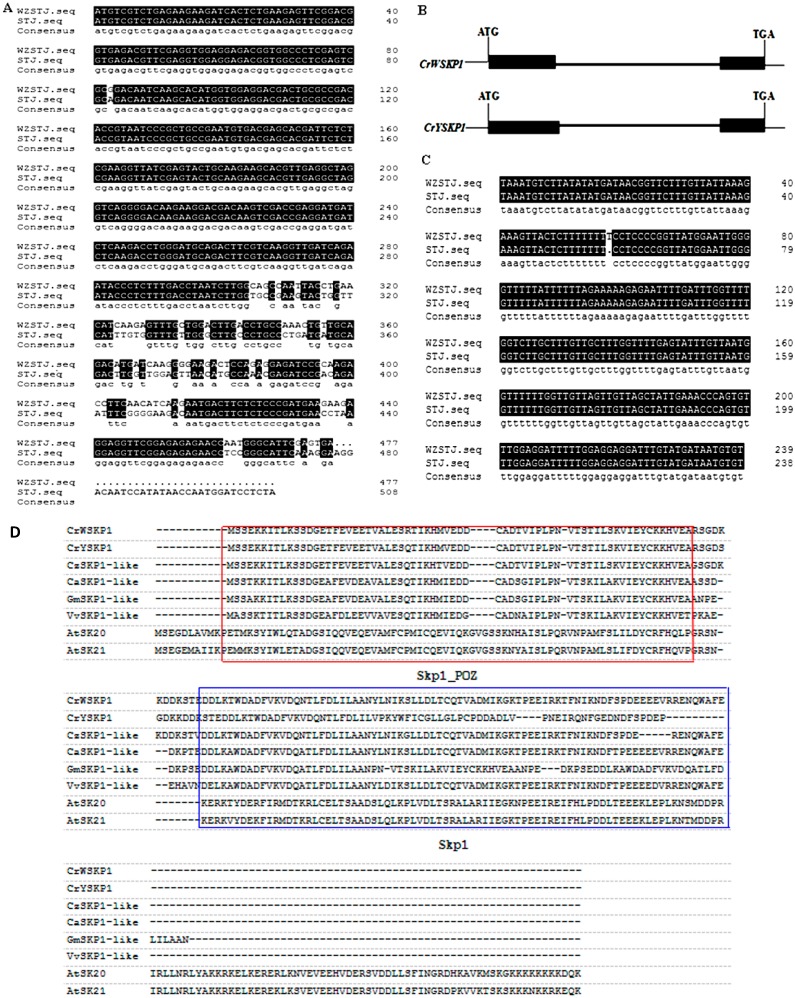
Analyses of *CrWSKP1* and *CrYSKP1* genes. (**A**) Alignments of cDNA sequences of *CrWSKP1* and *CrYSKP1* genes between “Wuzishatangju” (WZSTJ) and “Shatangju” (STJ); (**B**) Exon-introns structure of *CrWSKP1* and *CrYSKP1* genes. *CrWSKP1*, DNA sequence of the *Skp1-like* gene from “Wuzishatangju”; *CrYSKP1*, DNA sequence of the *Skp1-like* gene from “Shatangju”. Solid boxes indicate exons, and bold lines represent introns; (**C**) Alignments of partial DNA sequences of *CrWSKP1* and *CrYSKP1* genes between “Wuzishatangju” (WZSTJ) and “Shatangju” (STJ); (**D**) Alignments of the putative amino acid sequences of *CrWSKP1* and *CrYSKP1* genes from different plant species. *Cr*W*SKP1* (“Wuzishatangju”), *CrYSKP1* (“Shatangju”), *CzSSKP1-like* (*Citrus maxima*, ACP20181), *CaSKP1-like* (*Cicer arietinum*, XP004512164), *GmSKP1-like* (*Glycine max*, XP003517160), *VvSKP1-like* (*Vitis vinifera*, XP002279232), *AtSK20* (*Arabidopsis thaliana*, A8MQG7), *AtSK21* (*Arabidopsis thaliana*, Q8LF97). Higher conserved regions were marked in box with Skp1_POZ and Skp1.

The full-length cDNA sequence of *SKP1-like* gene from mutant “Wuzishatangju” mandarin (designated as *CrWSKP1*) was 779 bp and contained an open reading frame of 477 bp (Figure S1) encoding 158 amino acids. The ORF sequences of *Skp1-like* gene from its original cultivar “Shatangju” mandarin (designated as *CrYSKP1*) were 510 bp. Compared to “Shatangju”, there were 33 bases missing in the ORF of ‘Wuzishatangju’ (Figure 1A). The full-length of *CrWSKP1* genomic DNA from “Wuzishatangju” and “Shatangju” were, respectively, 1296 and 1294 bp with two exons and one intron (Figure 1B). There are 7 bases different in DNA sequences of *CrWSKP1* and *CrYSKP1* genes between “Wuzishatangju” and “Shatangju” mandarins (Figure 1C).

Sequence alignment showed that *CrWSKP1* and *CrYSKP1* genes had two highly conserved regions (Skp1_POZ and Skp1) and an intervening region between the two domains (Figure 1D). *CrWSKP1* shared the highest (99%) amino acid sequence identity with *CrYSKP1* gene.

### 2.2. Phylogenetic Analysis of SKP1-like Genes

**Figure 2:**
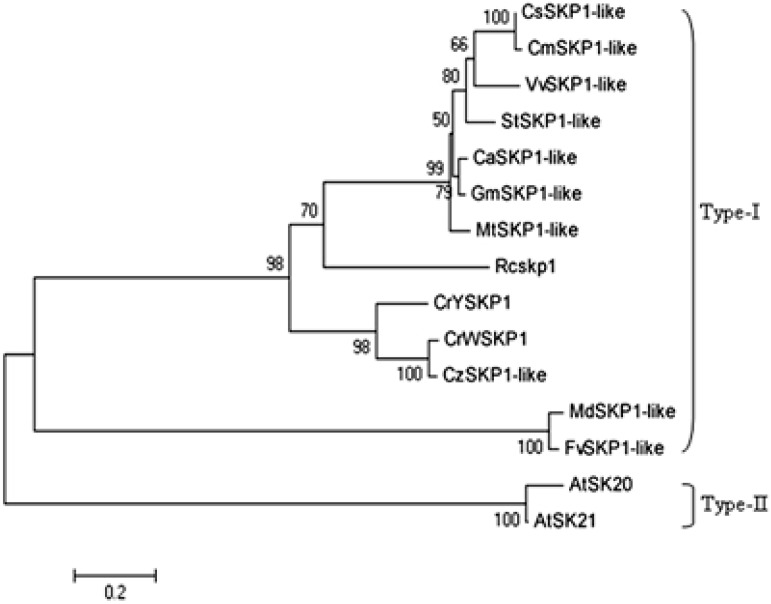
Phylogenetic relationship of *CrWSKP1* and *CrYSKP1* with *SKP1-like* genes from other plants. The numbers of each interior branch are the percentage bootstrap values (re-sampling). *CrWSKP1* (“Wuzishatangju”), *CrYSKP1* (“Shatangju”), *CzSKP1-like* (*Citrus maxima*, ACP20181), *CaSKP1-like* (*Cicer arietinum*, XP004512164), *GmSKP1-like* (*Glycine max*, XP003517160), *VvSKP1-like* (*Vitis vinifera*, XP002279232), *CsSKP1-like* (*Cucumis sativus*, XP004144851), *CmSKP1-like* (*Cucumis melo*, XP008447650), *StSKP1-like* (*Solanum tuberosum*, XP006362720), *MtSKP1-like* (*Medicago truncatula*, XP003612227), *RcSKP1* (*Ricinus communis*, XP002510577), *MdSKP1-like* (*Malus domestica*, XP008357122), *FvSKP1-like* (*Fragaria vesca* subsp. Vescal (XP004287783), *AtSK20* (*Arabidopsis thaliana*, A8MQG7), *AtSK21* (*Arabidopsis thaliana*, Q8LF97).

To determine the phylogenetic relationship of *CrWSKP1* and *CrYSKP1* with *SKP1*-*like* genes from the other plants, a phylogenetic tree was constructed using 15 *SKP1-like* amino acids sequences (Figure 2). As shown in Figure 2, those *SKP1-like* genes were classified into two types, *i.e*., Type I and Type II. *CrWSKP1* and *CrYSKP1* belonged to Type I. Cluster analysis revealed that *CrWSKP1* and *CrYSKP1* were more closely related to the *CzSKP1-like* (*Citrus maxima*, ACP20181). *CrWSKP1* shared the highest (99%) amino acid sequence identity with *CrYSKP1*, followed by *Citrus maxima* *CzSKP1-like* (ACP20181) (98%), *Cicer arietinum* *CaSKP1-like* (87%), *Glycine max* *GmSKP1-like* (85%), and *Vitis vinifera* *VvSKP1-like* (81%) (Figure 2).

### 2.3. Southern Blot Analysis of SKP1-like Genes

**Figure 3:**
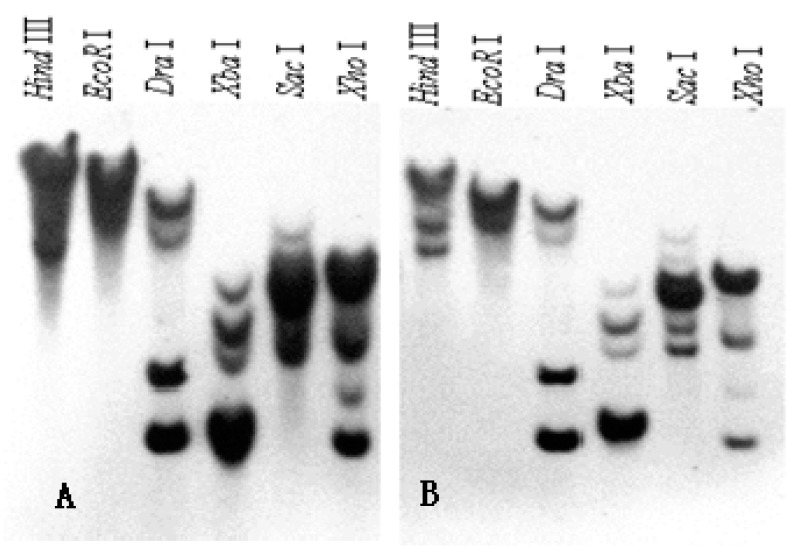
Southern analysis of *Skp1-like* genes in (**A**) “Wuzishatangju” and (**B**) “Shatangju” mandarins.

Southern blot was performed to determine whether *SKP1**-**like* represents a single or multi locus in the genome of “Wuzishatang” and “Shatangju” mandarins. As shown in Figure 3, multiple hybridization bands were observed when genomic DNA was digested with *Hin*d III, *Eco*R I, *Dra* I, *Xba* I, *Sac* I and *Xho* I, respectively. According to the result, we concluded that *Skp1-like* genes were encoded by a multiple-copy gene in “Wuzishatangju” and “Shatangju” genome.

### 2.4. Molecular Analyses of CrWSKP1 Transgenic Tobacco

Tobacco transformants containing *CrWSKP1* driven by CaMV 35S promoter were obtained by *Agrobacterium*-mediated transformation of leaf explants. The incorporation and expression of *CrWSKP1* in tobacco were confirmed by PCR (Figure S2), Southern blot (Figure S3A) and real-time PCR (Figure S3B) analyses. Four independent one-copy *CrWSKP1* tobacco lines were achieved (shown by red arrow) and used for further analyses. *CrWSKP1* expression was detected in all transgenic tobacco compared to no expression in wild type (WT) (Figure S3B).

### 2.5. Phenotype Analyses of CrWSKP1 Transgenic Tobacco

**Figure 4:**
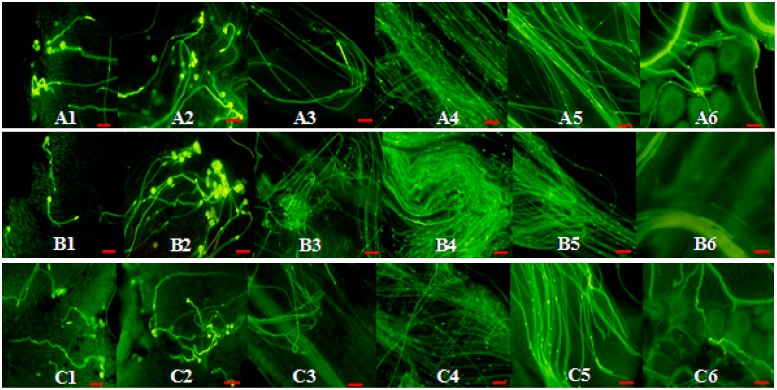
Pollination and fertilization of *CrWSKP1* transgenic tobacco at different stages S. (**A1**–**A6**) WT×WT; (**B1**–**B6**) *CrWSKP1*×*CrWSKP1*; (**C1**–**C6**), *CrWSKP1*×WT; 1, 2, 3, 4, 5, 6: 1, 2, 3, 4, 5, 6 represent days after self- and cross-pollination. Scale bar = 100 μm.

**Figure 5:**
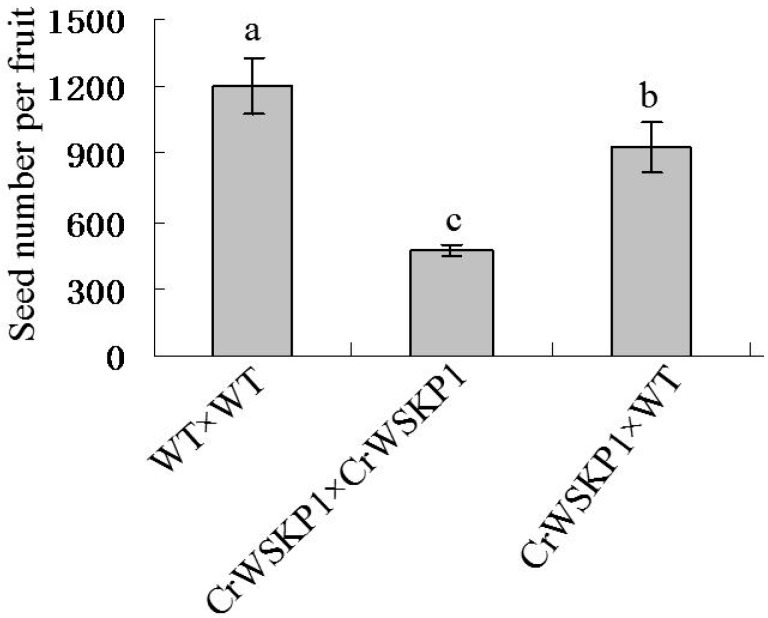
Seed number per fruit of *CrWSKP1* transgenic tobacco after self- or cross-pollination. The vertical bars represent standard deviation (±SD) of three biological replicates. a,b,c represent significant difference using the LSD test at *p* ≤ 0.05.

No significant difference in pollen viability and pollen germination rates was detected between CrWSKP1 transgenic tobacco and WT (Table S1). Three pollination combinations, namely WT×WT, *CrWSKP1*×*CrWSKP1*, *CrWSKP1*×WT were carried out to study the function of *CrWSKP1* gene in tobacco. After dyeing with staining agents, pollen grains became golden-yellow, pollen tubes became fluorescent green, and a bright spot of sperm and vegetative nuclei were detected at the apex of pollen tubes (Figure 4). Pollen grains germinated on the stigma at 1 day after pollination for all pollination combinations (Figure 4(A1–C1)). Pollen tubes entered into stigmas and grew downward through styles, and vascular bundles were observed around the pistillar chord at 2 days after self- and cross-pollinations (Figure 4(A2–C2)). Three days after self- and cross-pollinations, almost all pollen tubes elongated constantly in styles (Figure 4(A3–C3)). Compared to the other combinations, pollen tubes of self-pollinated *CrWSKP1*×*CrWSKP1* began to twist (Figure 4(B3)). Pollen tubes of WT×WT grew normally at 4 days after self-pollination (Figure 4(A4)) while approximately 85% pollen tubes of self-pollinated *CrWSKP1* transgenic tobacco became twisted at 4 days after self-pollination (Figure 4(B4)). Pollen tubes reached the bottom of style at 5 days after self- and cross-pollination for all combinations. The number of pollen tubes of self-pollinated *CrWSKP1*×*CrWSKP1* reaching the bottom of styles was significantly fewer than that of the other combinations (Figure 4(A5–C5)). Six days after self- and cross-pollinations, pollen tubes entered into ovaries and ovules through micropyles to complete fertilization (Figure 4(A6–C6)). Number of pollen tubes of self-pollinated *CrWSKP1*×*CrWSKP1* entered into ovules was significantly fewer than that of the other combinations. Compared to self-pollination of WT, seed number of transformants was significantly reduced after self-pollination of *CrWSKP1*×*CrWSKP1* and significantly increased after cross-pollination of *CrWSKP1*×WT (Figure 5). Those results suggested that the *CrWSKP1* gene is involved in the SI reaction of “Wuzishatangju”.

## 3. Discussions

*SKP1-like* gene contains a large number of family members that play key roles in cell-cycle progression, transcriptional regulation, flower formation, signal transduction, and many other cellular processes. *SKP1-like* genes have been characterized from *Arabidopsis* [22,27,34,35], rice (*Oryza sativa*) [36,37,38], wheat (*Triticum aestivum*) [26], *Antirrhinum* *hispanicum* [11], lily (*Lilium longiflorum*) [29], “Shatian” pummelo (*C. grandis* Osbeck), sweet cherry (*P.* *avium*) [30] and apple (*Malus*×*domestica*) [39]. Currently, twenty-one and thirty-one *SKP1-like* genes were identified from *Arabidopsis* and *Oryza sativa*, respectively, and they have pleiotropic effects. However, little information is available about them in citrus. Chai *et al.* [25] found that *CgSKP1* was closely related to flower development of “Shatian” pummelo. Our previous work suggested that *CrWSKP1* was obviously up-regulated in ovaries before pollination and in pistils at 4 days after self-pollination from “Wuzishatangju” [32]. In this study, full-length cDNA and DNA *SKP1-like* genes, designated as *CrWSKP1* and *CrYSKP1*, were isolated from *C*. *reticulata* Blanco cv. Wuzishatangju and Shatangju. *CrWSKP1* showed high amino acid sequence similarity with Shatangju (99%), *Citrus maxima* (98%), *Cicer arietinum* (87%), *Glycine max* (85%) and *Vitis vinifera* (81%) (Figure 2)*.* Multiple copies of *SKP1-like* genes existed in the genome of both “Wuzishatangju” and “Shatangju” suggesting that *SKP1-like* genes belong to a multigene family.

Skp1 is a small protein of approximately 160 amino acids. It contains two highly conserved domains (a Skp1_POZ domain, which interacts with Cul1 at the N terminus, and a Skp1 domain, which interacts with F-box domains at the C terminus) and an intervening region between them [25,30,40]. In our study, *CrWSKP1* comprised of 158 amino acids and contained both the Skp1_POZ and Skp1 domains (Figure 1C). The result is consistent with the structure and conserved domains of SKP1 protein [40]. Plant *SKP1* genes can be classified into type I and type II; type II is much longer than type I. In this study, deduced amino acid sequence of *CrWSKP1* was aligned with both type I and type II, and results showed that *CrWSKP1* had high similarity with the type I of “Shatian” pummelo *CgSKP1* [25], suggesting that *CrWSKP1* belonged to type I. Genomic sequence of the *CrWSKP1* gene contained two exons and one intron, a typical characteristic of type I [34,41]; a similar structure is also observed in “Shatian” pummelo [25], rice [38], and *Arabidopsis* [8].

Skp1 is a core component of the SCF complex mediating protein degradation by U/26S, which is involved in the SI reaction in model plants [42,43,44,45,46]. Kahloul *et al.* [38] found that these structures of *SKP1* proteins seemed to interact with most of the F-box proteins. The pollen-specific *AhSSK1*, an *Antirrhinum* *hispanicum* SLF-interacting *SKP1-like* gene, could be recruited exclusively as the adaptor of putative SCF in those plants with S-RNase-based SI [11,12]. Chang *et al.* [29] obtained three *SKP1-like* genes (LSK1-LSK3) from GSI lily (*Lilium longiflor*um) and found that they are specifically expressed in late pollen developmental stages and the elongating pollen tube. Lily pollen tubes harboring overexpressed *LSK* genes (LSKΔ) were substantially inhibited within the self-pollinated styles and almost all the LSK2Δ-GFP-transformed pollen tubes were arrested on the stigma. Those results suggest that *LSK2* and *LSK3* are involved in this complex GSI machinery of lily [29]. *PavSSK1*, a sweet cherry (*Prunus avium*) SFB (*PavSFB*)-interacting *Skp1-like* gene could be a functional component of the SCF complex involved in self-/nonself-recognition in the GSI of *Prunus* [30]. Citrus belongs to GSI and the underlying mechanism is still unclear. In the present study, we isolated *CrWSKP1* gene from SI Wuzishatangju mandarin and explored its function by transgenic experiments in tobacco. There was no significant difference in pollen viability and pollen germination rates between *CrWSKP1* transgenic tobacco and wild type (Table S1). Approximately 85% of pollen tubes of self-pollinated *CrWSKP1* transgenic tobacco became twisted at 4 days after self-pollination and pollen tubes of self-pollinated *CrWSKP1* transgenic tobacco reaching the bottom of style was significantly fewer than that of wild type (Figure 4(A5–C5)). Interestingly, this disturbance was more severe in self-pollinated *CrWSKP1* transgenic tobacco than that in cross-pollinated pollen tubes (Figure 4(B4,C4)). Compared to self-pollination of WT, seed number of *CrWSKP1* transgenic tobacco was significantly reduced whether they are self- or cross-pollination combinations (Figure 5). *CrSKP1* overexpression does not cause a general defect in pollen tube growth, directionality and/or guidance. In theory, the cross *CrWSKP1*×WT have the same seed number as the cross WT×WT. In this study, we found that some pollen tubes became twisted and could not enter ovaries at 3 days after cross-pollinated *CrWSKP1*×WT. Therefore, cross *CrWSKP1*×WT does not have the same seed number than that of WT×WT (Figure 5). However, our results did not clarify whether *CrWSKP1* is the cause of SI reaction of “Wuzishatangju” through the SCF complex mediating protein degradation by U/26S. Further work on identifying protein interacting with CrWSKP1 is necessary to elucidate its crucial role in the SI reaction of “Wuzishatangju”.

## 4. Experimental Section

### 4.1. Plant Materials

Mutant “Wuzishatangju” (SI) and its original cultivar “Shatangju” (SC) mandarins were grown in the same orchard of South China Agricultural University and used as plant materials. Leaves from aseptic tobacco (*Nicotiana tabacum* L. cv. W38) plantlets cultured on MS medium [47] were used as the source of transformation explants.

### 4.2. Genomic DNA and Total RNA Extraction

Genomic DNA was isolated using a CTAB method [48]. Total RNA was extracted using the RNAout kit (TIANDZ, Beijing, China), and pretreated by RNase-free DNase I (TaKaRa, Dalian, China). Qualities and concentrations of RNA and DNA were determined using a spectrophotometer (Bio-Rad Laboratories, Hercules, CA, USA) and 1.2% (*w*/*v*) agarose gel electrophoresis. The first strand cDNA was synthesized according to the manufacturer’s instructions using a cDNA synthesis kit (TaKaRa).

### 4.3. Cloning and Sequence Analysis of Skp1-like Gene

A *SKP1-like* coding sequence (CDS) was obtained through SSH libraries of 72 h styles after self-pollination of “Wuzishatangju” and cross-pollination of “Wuzishatangju”×“Shatangju” [32]. A pair of primers (*SKP1*-F and *SKP1*-R) (Table 1) was used to amplify the full-length DNA and cDNA of *CrWSKP1* and *CrYSKP1* genes including start and stop codons.

**Table 1:** Primer sequences and PCR procedures used in this study.

Primer Name	Primer Sequences (5′–3′)	Procedures of PCR
SKP1-F	GAAACGATGTCGTCTGAGAAGAAGAT	94 °C 4 min; 94 °C 30 s, 59 °C 30 s, 72 °C 60 s, 35 cycles; 72 °C 10 min
SKP1-R	GTCCTTCACTCGAATGCCCATTGGTT	
TZSKP1-F	ATAAAATGAGAACTTAATTTAC	94 °C 4 min; 94 °C 30 s, 57 °C 30 s, 72 °C 60 s, 35 cycles; 72 °C 10 min
TZSKP1-R	TCACTCGAATGCCCATTGGTT	
NPTII-F	GTTCTTTTTGTCAAGACCGACC	94 °C 4 min; 94 °C 30 s, 55 °C 30 s, 72 °C 60 s, 35 cycles; 72 °C 10 min
NPTII-R	CAAGCTCTTCAGCAATATCACG	
35S-F	GAGGACCTAACAGAACTCG	94 °C 4 min; 94 °C 30 s, 57 °C 30 s, 72 °C 60 s, 35 cycles; 72 °C 10 min
35S-R	GTCTTGCGAAGGATAGTGG	
RTSKP1-F	ATGTCGTCTGAGAAGAAGATC	94 °C 5 min; 94 °C 30 s, 59 °C 30 s, 72 °C 1 min, 35 cycles; 72 °C 10 min
RTSKP1-R	CTCGAATGCCCATTGGTTCTC	
ChvA-F	TCCATCAGCAACGTGTCGGTGCT	94 °C 4 min; 94 °C 30 s, 60 °C 30 s, 72 °C 90 s, 35 cycles; 72 °C 10 min
ChvA-R	GTGGAAAGGCGGTGAGCGATGAT	
QSKP1-F	CTCTTTGACCTAATCTTGGCAG	95 °C 1 min; 95 °C 15 s, 55 °C 20 s, 72 °C 30 s, 40 cycles
QSKP1-R	CTTGCGGATCTCCTCTGGAGTC	
Q-actin-F	CTGGCATTGCAGATCGTATGA	95 °C 1 min; 95 °C 15 s, 55 °C 20 s, 72 °C 30 s, 40 cycles
Q-actin-R	GCGCCACCACCTTGATCTT	

Nucleotide sequences of *CrWSKP1* and *CrYSKP1* genes were analyzed using the NCBI Blast program [49]. Amino acid sequence analysis and a homology tree were constructed using MEGA software (Version 5.05) (Arizona State University, Tempe, AZ, USA). The number for each interior branch is the percent bootstrap values calculated from 1000 replicates.

### 4.4. Southern Blot Analysis

Genomic DNA (10.0 μg per sample) from “Wuzishatangju” and “Shatangju” mandarins was digested in separate tubes with *Hin*d III, *Eco*R I, *Dra* I, *Xba* I, *Sac* I and *Xho* I overnight at 37 °C, separated on a 0.8% (*w*/*v*) agarose gel and transferred onto Hybond-N^+^ nylon membranes (Hybond N^+^, Amersham Biosciences Corp, NJ, USA) [50]. Probes (353 bp) from a partial region of the *CrWSKP1* gene for hybridization were prepared from PCR product using TZSKP1-F and TZSKP1-R primers (Table 1) and used in DIG-dUTP according to the manufacturer’s instruction (Roche Applied Science, Mannheim, Germany). Pre-hybridization, hybridization, and detection of the labeled probe were performed according to Miao *et al.* [51].

### 4.5. Genetic Transformation of Tobacco

To construct the *CrWSKP1* expression vector, the *CrWSKP1* fragments were excised using *Bam*H I and *Sac* I and inserted into pBI121 vector digested with the same two enzymes. The constructed expression vector was named pBI121-CrWSKP1 under control of the CaMV 35S promoter. The recombinant plasmids were transferred into *Agrobacterium tumefaciens* strain EHA105 by the liquid nitrogen freeze-thaw method.

Putative transformants were screened by PCR analysis using NPT II, 35S, RTSKP1 and ChvA primers (Table 1). T_0_ positive-PCR transformed lines were further confirmed by Southern blot. For Southern hybridization, 20.0 μg of genomic DNA was digested with *Eco*R I (cloning site for *CrWSKP1* gene in pBI121-CrWSKP1 vector), blotted, and probed by using the NPT II gene according to the protocol of Miao *et al.* [51].

Expression levels of *CrWSKP1* in single-copy T_0_ transgenic lines were assayed by real-time PCR using the SYBR ExScript RT-PCR Kit (TaKaRa). Q-Actin*-*F and Q-Actin*-*R primers were used to amplify the tobacco *actin* gene (accession No. U60491.1) as a loading control to normalize samples in separate tubes. Quantitative real-time PCR were performed in triplicate for each sample using the primers of QSKP1-F and QSKP1-R (Table 1). The expression levels of the *CrWSKP1* gene were calculated using the 2^−∆∆*C*t^ method [52].

### 4.6. Pollination and Fertilization of Transgenic Tobacco

To explore function of *CrWSKP1* gene in transgenic tobacco, mature pollen grains from single-copy transgenic lines and wild types (WT) were collected to assay pollen viability and pollen germination rate according to Ye *et al.* [1]. Before artificial-pollination, the flowers were emasculated. Pollen was gently smudged with a brush on the stigma and then quickly bagged. Pistils, including stigmas, styles and ovaries, were collected at 1, 2, 3, 4, 5 and 6 days from single-copy self-pollinated transgenic *CrWSKP1* lines×transgenic *CrWSKP1* lines (*CrWSKP1*×*CrWSKP1*), cross-pollinated transgenic *CrWSKP1* lines×WT (*CrWSKP1*×WT) using self-pollinated WT×WT as control. Sections were made following the procedures of Shi and Hou [53] and Tao *et al.* [54]. Pollen tubes were stained with 0.1% aniline blue (Sigma, St. Louis, MO, USA) for 30 min. Pollination and fertilization of all combinations were observed with a fluorescent microscope (OLYMPUS BH_2_-RFCA, Olympus Optical Co., Ltd., Tokyo, Japan) and photos were obtained by a digital microphotograph system (OLYMPUS DP70, Olympus Optical Co., Ltd., Tokyo, Japan). Mature seed number per fruit of *CrWSKP1* transgenic tobacco was collected after self- or cross-pollination combinations.

### 4.7. Statistical Analysis

Statistical analyses were performed using DPS 7.05 software (Zhejiang University, Hangzhou, Zhejiang, China). Differences between compared sets were considered significantly using the LSD test at *p* ≤ 0.05.

## 5. Conclusions

We cloned a *CrWSKP1* gene from “Wuzishatangju” mandarin with high similarity to the other plant *SKP1-like* genes. Approximately 85% of pollen tubes of self-pollinated *CrWSKP1* transgenic tobacco plants became twisted at 4 days after self-pollination. Pollen tubes of self-pollinated *CrWSKP1* transformants entering into ovule were significantly fewer than that of the control. Seed number of self-pollinated *CrWSKP1* transformants was significantly reduced. Those results suggested that the *CrWSKP1* gene is involved in the SI reaction of “Wuzishatangju”. Further work using yeast two-hybrid analysis is needed to identify proteins interacting with *CrWSKP1* and to then study their *in vivo* function in citrus or other model plant systems.

## Supplementary Materials

Supplementary materials can be found at http://www.mdpi.com/1422-0067/16/09/21695/s1.
