# Supplementary material for: CrWSKP1, an SKP1-like Gene, Is Involved in the Self-Incompatibility Reaction of “Wuzishatangju” (Citrus reticulata Blanco)
Source: Int J Mol Sci. 2015 Sep 9;16(9):21695–710. doi: 10.3390/ijms160921695 (PMC4613275; doi:10.3390/ijms160921695)
Supplement: Supplementary file 1 [file ijms-16-21695-s001.pdf]

Supplementary Information

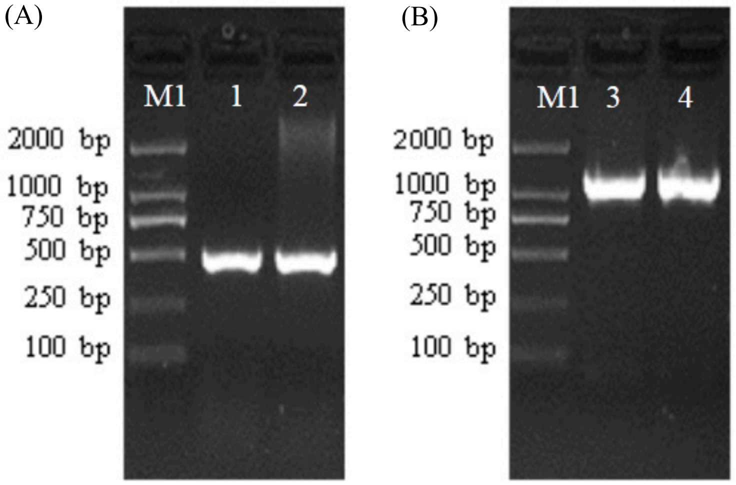

Figure S1. PCR products of full-length cDNA (**A**) and DNA (**B**) of *CrWSKP1* and *CrYSKP1* genes from "Wuzishatangju" and "Shatangju" mandarins. M1, DL2000 marker; 1, full-length cDNA of *CrWSKP1* genes from "Wuzishatangju"; 2, full-length cDNA of *CrYSKP1* genes from "Shatangju"; 3, full length genomics DNA of *CrWSKP1* genes from "Wuzishatangju"; 4, full length genomics DNA of *CrYSKP1* genes from "Shatangju".

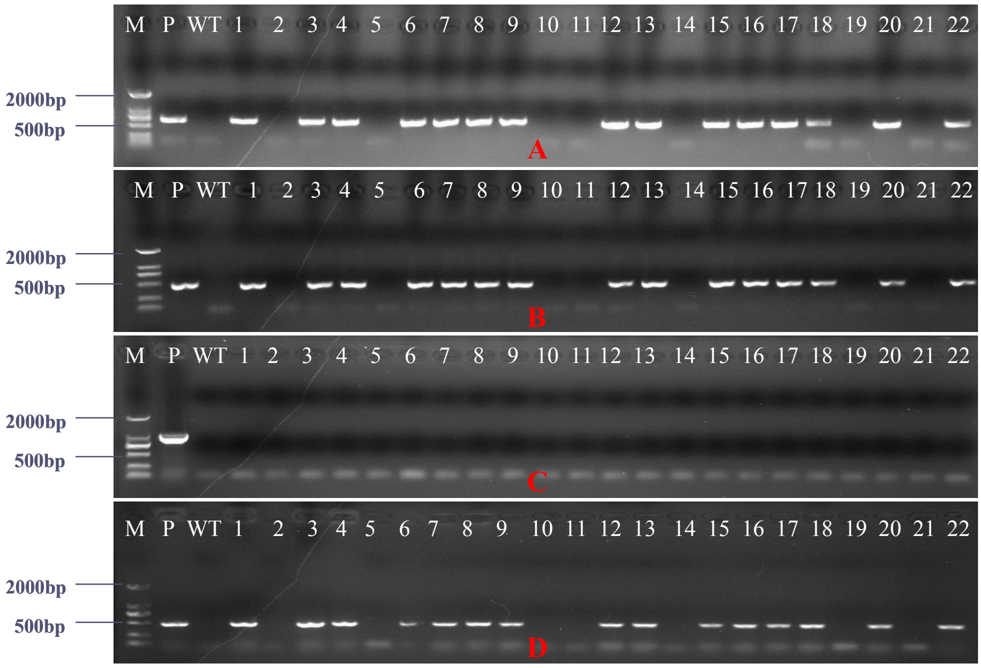

Figure S2. PCR analyses of Kan-resistant tobacco plants harboring *CrWSKP1* gene. (**A**) NPT II primers; (**B**) 35S primers; (**C**) ChvA primers; (**D**) *CrWSKP1* primers; M, Marker DL2000; WT, wild type; P, positive control; 1–22, Kan-resistant tobacco plants.

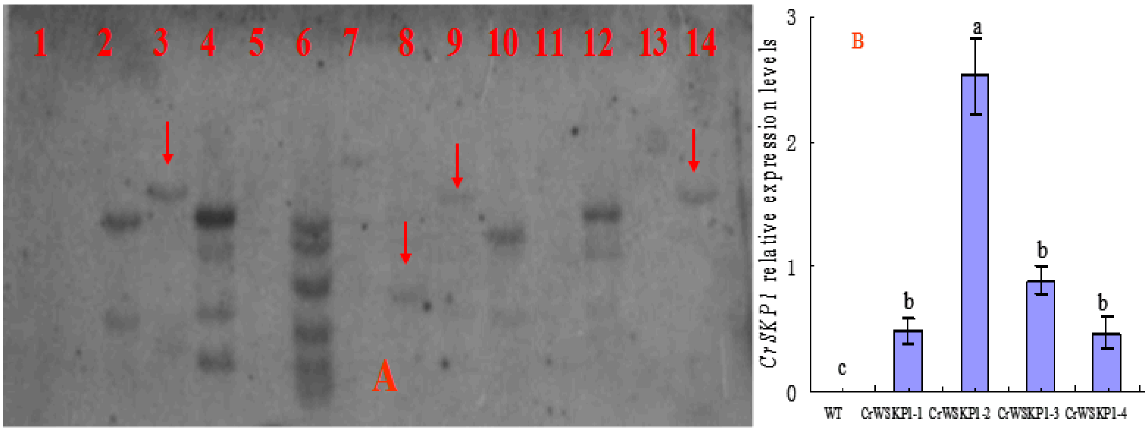

Figure S3. Southern blot analyses *CrWSKP1* in transformed tobacco plants (**A**) and expression analyses of *CrWSKP1* in different transgenic tobacco lines (**B**) 1–14, different PCR-positive tobacco plants. Red arrow indicated four independent one-copy *CrWSKP1* tobacco lines. a, b represent significant difference using the LSD test at $p \leq 0.05$.

Table S1. Pollen viability and pollen germination rate of *CrWSKP1* transgenic tobacco.

Types	Pollen Viability (%)	Pollen Germination Rate (%)
CrWSKP1 transgenic tobacco lines	77.1 a	64.7 a
Wild type (control)	81.3 a	67.8 a

a means within a column followed by the same letter were not significantly different using the LSD test at $p \leq 0.05$.
